# Superior anti-pulmonary viral potential of *Natrialba* sp. M6-producing surfactin and C50 carotenoid pigment with unveiling its action modes

**DOI:** 10.1186/s12985-023-02215-8

**Published:** 2023-10-30

**Authors:** Ghada E. Hegazy, Marwa M. Abu-Serie, Nadia A. Soliman, Mohamed Teleb, Yasser R. Abdel-Fattah

**Affiliations:** 1 National Institute of Oceanography and Fisheries, NIOF, Cairo, Egypt; 2 Medical Biotechnology Department, Genetic Engineering and Biotechnology Research Institute (GEBRI), City of Scientific Research and Technological Applications, Alexandria, Egypt; 3 Bioprocess Development Department, Genetic Engineering and Biotechnology Research Institute (GEBRI), City of Scientific Research and Technological Applications, Alexandria, Egypt; 4 Department of Pharmaceutical Chemistry, Faculty of Pharmacy, Alexandria University, Alexandria, Egypt

## Abstract

**Background:**

Respiratory viruses, particularly adenoviruses (ADV), influenza A virus (e.g., H1N1), and coronaviruses (e.g., HCoV-229E and SARS-CoV-2) pose a global public health problem. Therefore, developing natural wide-spectrum antiviral compounds for disrupting the viral life cycle with antioxidant activity provides an efficient treatment approach. Herein, biosurfactant (Sur) and C50 carotenoid pigment (Pig) of haloalkaliphilic archaeon Natrialba sp. M6 which exhibited potent efficacy against hepatitis and anti-herpes simplex viruses, were investigated against pulmonary viruses.

**Methods:**

The cytotoxicity of the extracted Sur and Pig was examined on susceptible cell lines for ADV, HIN1, HCoV-229E, and SARS-CoV-2. Their potential against the cytopathic activity of these viruses was detected with investigating the action modes (including, virucidal, anti-adsorption, and anti-replication), unveiling the main mechanisms, and using molecular docking analysis. Radical scavenging activity was determined and HPLC analysis for potent extract (Sur) was performed.

**Results:**

All current investigations stated higher anti-pulmonary viruses of Sur than Pig via mainly virucidal and/or anti-replicative modes. Moreover, Sur had stronger ADV’s capsid protein binding, ADV’s DNA polymerase inhibition, suppressing hemagglutinin and neuraminidase of H1N1, and inhibiting chymotrypsin-like (3CL) protease of SARS-CoV-2, supporting with in-silico analysis, as well as radical scavenging activity than Pig. HPLC analysis of Sur confirmed the predominate presence of surfactin in it.

**Conclusion:**

This study declared the promising efficacy of Sur as an efficient pharmacological treatment option for these pulmonary viruses and considered as guide for further in vivo research.

## Background

Acute pulmonary infections are a serious public health concern and a leading contributor of morbidity and mortality around the world, mostly affecting young children and elderly individuals. Several viruses attack the human respiratory tract, causing symptoms ranging from mild upper airway involvement to life-threatening acute respiratory distress syndrome (ARDS). The main respiratory viruses are adenoviruses (ADV), influenza A virus (orthomyxoviruses), and coronaviruses (CoVs). Pandemic viruses are mostly RNA genome, such as influenza (H1N1 in 2009) and coronaviruses (SARS-CoV-2 in 2019) inducing apoptosis-dependent cell death, while ADV (non-enveloped DNA genome) cause direct cell lysis [1].

Adenovirus is one of the most important pathogens responsible for acute respiratory distress syndrome. There are over 70 ADV serotypes. A previous study illustrated that ADV7 is responsible for highly severe adenoviral diseases in children. Unfortunately, specific and direct anti-ADV drugs are currently not available [2]. ADV attaches to host cell receptors via its fiber-pentose complex, and once it is engaged, the viral fiber protein dissociates from the capsid, and its pentose base binds to the integrin entry receptor, resulting in virus endocytosis [3]. After ADV entry into the nucleus for replication with the assistance of ADV DNA polymerase, new virions are assembled. These new viral progenies are released by cell lysis (direct host cell membrane damage) [4]. H1N1 starts the replicative cycle by binding its hemagglutinin to sialic acid on ciliated epithelial lung cells mediating endocytosis into host cells. Following replication, the ribonucleocapsids engage with hemagglutinin and neuraminidase, and after membrane depolarization, viral budding and host cell release begins via neuraminidase enzymatic action to infect new cells, causing lung tissue damage [1, 4].

A high mortality rate is associated with the Coronaviridae family, which includes seven human species (HCoV-229E, HCoV-HKU1, HCoV-OC43, HCoV-NL63, MERS-CoV, SARS CoV, and SARS-CoV-2). This CoV family is classified mainly into two serologic and genetic groups (alphacoronavirus genera “e.g., HCoV-229E” and betacoronavirus genera “e.g., the most pandemic SARS-CoV-2”) [1, 5]. CoVs have a conserved genomic architecture and share the same replication cycle. This cycle is initiated by the engagement of the binding domain (RBD) of spike protein (HCoV-229E and SARS-CoV-2) with the host cell receptors (human aminopeptidase N and angiotensin converting enzyme (ACE)2, respectively) [1, 5]. Following endocytosis, CoV genome is released into cytoplasm, and part of the genomic RNA is translated into two polyproteins containing all non-structural proteins (nps, including RNA replicase-transcriptase complex-mediated CoV replication), which are primarily generated by chymotrypsin-like (3CL) protease action. This main protease’s cleavage sites and substrate specificity are highly conserved among CoVs [1, 5–7]. The SARS-CoV-induced dysregulation of the renin-angiotensin system is primarily responsible for the worsening tissue damage and proinflammatory response [1]. The latter propagate viral infection-mediated tissue damage from mild or moderate to severe by overproduction of cytotoxic free radicals (reactive oxygen (ROS) and nitrogen species (NOS)) [1].

A prolonged viral pulmonary infection without treatment leads to organ failure and death. Unfortunately, because of the recurring mutations that lead to the persistent generation of new resistant viral strains, there is only a limited number of antiviral drugs currently available against these viruses [1, 4]. Therefore, developing effective and safe broad-spectrum antiviral agents affecting directly virus life cycle (entry and/or replication) with antioxidant activity may offer a better therapeutic strategy by halting the uncontrolled radical generation from causing the extensive cell damage.

Our recent studies revealed potent anti-hepatitis viral activity (HCV and HBV) of biosurfactant (Sur) and C50 carotenoid pigment (Pig) extracts of haloalkaliphilic archaeon *Natrialba* sp. M6, via inhibiting viral RNA polymerase and viral DNA polymerase, respectively [8, 9]. Sur also had high reactivity against viral envelops (HCV E2 and herpes simplex virus (HSV) glycoprotein D) as well as effective inhibition of HSV DNA polymerase at IC_50_ < 5 µg/mL [9]. *Natrialba* sp. M6 (source of these active extracts) is environmentally friendly and cost-effective microorganism that can adapt to a harsh hypersaline environment, producing numerous active metabolites including, a unique Sur agent and a rare Pig containing four hydroxyl groups (C50 bacterioruberin) [8, 9]. This carotenoid pigment has numerous health benefits, including anticancer, inhibition of viral replication [8], and prevention of COVID 19 complications (uncontrolled proinflammatory cytokine storm and dysregulating renin-angiotensin system) [10, 11]. Regarding natural biosurfactant, it is more effective than its synthetic counterpart in the term of decreasing ARDS complications. Noteworthy, alveolar type II epithelial cells synthesize surfactant in order to prevent collapse at the end of expiration by lowering air–liquid surface tension [12, 13]. Moreover, this secreted surfactant plays a key role in ameliorating lung damage by its anti-inflammatory potential and host defense against lung infections by regulating immune response and neutralizing viruses [12]. These viruses can impede with surfactant synthesis and secretion, resulting in suppression of surfactant production, alveolar collapse and ARDS. The latter is the most well-known surfactant deficiency. Therefore, treatment with exogenous surfactant components improved host’s anti-viral response as well as lung function [12, 13]. However, it is unknown whether pulmonary surfactant could defend against any lung viruses Therefore, in this current investigation, cytotoxicity of both extracted compounds on different susceptible cell lines and their antiviral potential on ADV, H1N1, HCoV-229E, and SARS-CoV-2 were measured with the elucidating their modes of action (direct virucidal, anti-adsorption, and anti-replication). This study was supported with molecular docking for the most effective antiviral mode(s) employing the Molecular Operating Environment (MOE) software [14]. Furthermore, their scavenging activity for oxygen and nitrogen radical species (ONRS) which mediate complications of viral infections, was assessed.

## Methods

### Isolation, enrichment and identification of Haloalkaliphilic *Natrialba* sp. M6

Collect water and sediment samples from different sites in El-Hamra lake, Wadi El-Natrun known for their haloalkaline conditions. Use sterile containers to ensure the integrity of the samples, a culture medium suitable for the growth of haloalkaliphilic archaea was followed (g/L): casamino acids, 5; KH_2_PO_4_, 1; MgSO_4_·7H_2_O, 0.2; NaCl, 200; trace metals, 1 mL; and Na_2_CO_3_, 18. Te trace metal solution contained (g/L) ZnSO_4_·7H_2_O, 0.1; MnCl_2_·4H_2_O, 0.03; H_3_BO_3_, 0.3; CoCl_2_·6H_2_O, 0.2; CuCl_2_·2H_2_O, 0.01; NiCl_2_·6H_2_O, 0.02; and Na_2_MoO_4_·H_2_O, 0.03 as described by [8, 9]. The medium should have high salt concentration (NaCl) and a pH range of 9–11. Adjust the pH using sodium carbonate (Na_2_CO_3_). Inoculate a small volume of the collected water and sediment samples into the prepared enrichment culture medium. Incubate the cultures at the desired temperature (typically around 37 °C) for several days to allow the growth of haloalkaliphilic archaea. Transfer a small volume of the enriched culture to a fresh batch of the culture medium to promote the growth of haloalkaliphilic archaea. Repeat this subculturing process several times to obtain a pure and distinct culture of haloalkaliphilic archaea. For testing the isolates ability for the production of biosurfactants, pre-culture of the archaeal isolates was prepared and the cell free supernatant adjusted for the following methods: emulsification index, oil spreading technique, hemolytic activity, and surface tension measurement [9]. The most promising biosurfactant producing isolate was chosen and molecularly identified using molecular techniques (DNA sequencing).

### Recovery of the Sur

The culture supernatant of *Natrialba* sp. M6, is subjected to centrifugation at 15,000 rpm for 30 min. Centrifugation separates the cells and other debris from the supernatant, allowing for the extraction of Sur. To facilitate the extraction process, the cell-free culture supernatant is acidified by adding concentrated hydrochloric acid (HCl) until the pH reaches 2, acidification helps to modify the ionic state and solubility of the biosurfactant, aiding its subsequent extraction. After acidification, the treated supernatant is stored at 4 °C overnight. This incubation period allows the Sur to precipitate and facilitates its separation from the remaining components of the culture supernatant.

The Sur residue in the precipitate was desiccated, weighted and dissolved in known volume from 0.1 M sodium bicarbonate [9, 15].

### Recovery of the Pig

The Pig produced by *Natrialba* sp. M6 can be extracted by the centrifugation of 50 ml of the culture broth at 10,000 rpm for 30 min at 4 °C. The supernatant was spun down, and 50 ml of distilled water was added to the clean pellets and then stored at 4 °C overnight to rupture the cells. A mixture of the solvents methanol-acetone (3:7 v/v) containing 0.1% butylhydroxytoluene (BHT) as antioxidant was used. This step was repeated until both the pellets and the solvents were becoming colorless, and then again centrifuged. The extracted Pig in solvent was stored at 45 °C overnight until solvent completely evaporated, and the Pig was collected, weighted and wrapped with aluminium foil to prevent light damage. The colored Pig solution was scanned by their absorbance in the wave length region ranged 200–700 nm. the highest wave length of the absorption was (λ350nm) by using coefficient value of absorption of 266,030 [8]**.**

### HPLC identification of the extracted Sur

HPLC system equipped with a Chromolith high performance RP-18 (100 * 4.6 mm, 5 µm) column was maintained and operated at 25 °C. The mixture of mobile phase consisting of a 3.8 mM TFA solution and an CAN (ratio of 20:80) were driven with a flow rate of 2.2.mL/min in an isocratic mode. The surfactin volume injection was set at 30 µL, and recorded by the detector device VDW at 205 nm. Each analysis run was done within 8 min. Methanolic surfactin standard stock has been prepared at concentration 5000 mg/L. Later, a surfactin solution series of different concentrations ranged from 10 to 1000 mg/L were prepared by the stock solution dilution with solvent methanol and then they have been stored at temperature 4ºC before using. The solution of a 3.8 mM TFA has been prepared in a 1 L deionized water volumetric flask and vortexed until the dissolution has been completed. Finally, an CAN and the TFA solution have been filtered via 0.22 µm filters of nylon and the gasses were removed before using [8].

### Cytotoxicity of Sur and Pig on viral host cell lines

The cytotoxicity of Sur and Pig extracts was assessed on three established cell lines, green monkey kidney epithelial (Vero), Madin-Darby canine kidney (MDCK), and Vero Clone E6 (Vero E6), which are susceptible to ADV7, H1N1, and coronaviruses, respectively. Cell viability was assessed by the crystal violet assay [16]**.** These permissive host cell lines were cultivated in DMEM containing 10% fetal bovine serum (FBS). After trypsinization, these cell lines (10^4^ cells/well) were cultured in 96-well cell culture plates and placed in 5% CO_2_ incubator at 37 °C. The next day, suspensions of Sur or Pig at different serially-diluted were incubated with these viral host cells for 72 h, in 5% CO_2_ incubator. After washing the cells with phosphate buffer saline (PBS) and fixation, cells were stained with 0.03% crystal violet for 10 min and then elution solution (0.9% sodium citrate and 1N HCl in ethanol) was added. Using ELISA reader (BMG LabTech, Germany), the absorbances were measured at 540 nm. The cell viability (%) was estimated in order to calculate the effective concentrations (EC_50_ and EC_100_) at which 50% and 100% cell viability, respectively, using Graphpad Instat software.

### Investigation of anti-ADV7 activity

#### Determination of inhibition potency on cytopathic activity of ADV7

The virus inoculum (10^–5^) was seeded to 96 well culture plate containing Vero cells monolayer and incubated in 5% CO_2_ incubator for 2 h at 37 °C. Then, the unabsorbed viruses in supernatant were removed and replaced with fresh culture medium containing serially-diluted concentrations of sur or pig. Then these cells were incubated in 5% CO_2_ incubator for 72 h at 37 °C. All wells (infected-untreated, healthy, treated infected cells) were stained with crystal violet stain as described above, to calculate % reduction of the infected cells lysis associated to exposure to Sur or Pig. The dose (IC_50_), at which 50% inhibition of virus-mediated cell lysis, was calculated by the Graphpad Prism software.

#### Crystal violet assay and quantitative PCR analysis for investigating the action modes

To investigate the direct virucidal effect, serially-diluted concentrations of Sur or Pig were incubated in 5% CO_2_ incubator for 2 h at 37 °C with 10^–5^ ADV7 before inoculating this treated suspension into a susceptible and permissive monolayer of Vero cells and further incubated for 2 h. Meanwhile, the anti-adsorption effect was measured by pretreating host monolayer of Vero cells with different concentrations of Sur or Pig for 2 h, then removing the supernatant and adding a suspension of infectious viruses to the monolayer for another 2 h of incubation. Concerning the anti-replicative effect, different diluted concentrations of Sur or Pig was added after incubating their host cells with ADV7 for 2 h and then supernatant removed. Then, the untreated and treated cells were stained with crystal violet, as described above, for assessing IC_50_ by the Graphpad Prism software.

For more accurate confirmation, the same conditions of three experiments of action modes were performed at lower IC_50_ (3 µg/mL), and then the treated and untreated infected host cells were collected for determining viral genome load using TaqMan-based real time PCR [17]**.** The used ADV7 primers were 5′-GAGGAGCCAGATATTGATATGGAATT-3′ and 5′-AATTGACATTTTCCGTGTAAAGCA-3′ with the probe 5′-6-carboxyfluorescein (FAM)-AAGCTGCTGACGCTTTTTCGCCTGA-6-carboxytetramethylrhodamine (TAMRA)-3′. Reaction mix contained Taq polymerase enzyme (0.05 U/L), the reaction buffer (250 nM probe, 0.4 mM dNTP, 400 nM reverse/forward primers and 4 mM MgCl_2_). PCR program was started at temperature 95 °C for 5 min next by 45 cycles at temperature 95 °C for 10 s, 55 °C for 30 s and then 72 °C for 20 s. Viral load was calculated using standard curve of the virus.

#### ELISA assessment of Sur or Pig binding to capsid protein of ADV7

Briefly, polystyrene plates were coated with recombinant antibody of ADV fiber (5 μg/ml). After overnight incubation, plates were washed and blocked with borate buffer saline containing skim dry milk then the preincubated mixture of 0.8 μg/ml of Sur or Pig and ADV was added. After 1 h incubation, wells were washed to remove unbinding mixture. Alkaline phosphatase-conjugated secondary antibody was added, followed by washing and adding p-nitrophenyl phosphate. The reaction was stopped by adding 3 N NaOH, and the absorbance was measured at wave length 405 nm.

#### Inhibitory effect on polymerase activity of ADV7

The inhibitory potential of Sur or pig on ADV by performing DNA polymerase activity using the method of [18]**.** Briefly, Sur or Pig serially-diluted concentrations were added to a reaction mixture of 7 mM MgCl_2_, 10 mM DTT, 25 mM Tris–HCl pH 7.8, 1 μg aphidicolin, activated DNA and 40 μM deoxynucleotides with 1 µCi radiolabeled [α-^32^P]dATP. After 1 h of incubation, the synthesized DNA was transferred to the disks of filter paper and then precipitated using trichloroacetic acid and then measured using a scintillation counter.

### Investigation of anti-H1N1 activity

#### Determination of inhibiting potency on cytopathic activity of H1N1

One day before viral infection, MDCK cells were seeded at a density of 2 × 10^4^cells/well into a 96-well culture plate. After cell attachment for 24 h. The cells were washed with PBS after removing the culture medium and then incubated with 100 µl of diluted H1N1 suspension containing CCID50 (50% of cell culture infective dose, 10^–5^). Then serially-diluted concentrations of Sur or Pig were added to the infected cells, exception of wells serving as positive infected (untreated) control. After 72 h incubation of culture plates at 37 °C in 5% CO_2_, cells were washed, fixed and stained with a 0.03% crystal violet solution as described above. The inhibitory dose at 50% cytopathic effect (IC_50_) was estimated by the Graphpad Prism software.

#### Investigation of anti-H1N1 action modes

To investigate the virucidal effect, serially-diluted concentrations of Sur were incubated with H1N1 for 1 h, in 5% CO_2_ incubator, with 10^–5^ H1N1, followed by 1 h incubation of this mixture (H1N1 + Sur) with monolayer of MDCK cells. The anti-adsorption effect was assessed by preincubating host cells with culture medium containing different concentrations of Sur for 1 h, then replacing this medium with H1N1 inoculum and incubating cells for 1 h. Regarding anti-replicative effect, serially-diluted concentrations of Sur were incubated with H1N1-infected MDCK cells for 1 h. In three plates of these three experiments, the infected medium was replaced by new culture medium that was incubated with cells for 72 h. Following that, all cells were stained with crystal violet, as demonstrated above, for estimation of IC_50_ by the Graphpad Prism software.

#### Hemagglutination inhibition assay

Hemagglutination inhibition (HI) assay was used to evaluate virucidal effect of Sur on H1N1 hemagglutinin for preventing its adsorption to erythrocytes [10, 19]. Erythrocytes were isolated by centrifugation of heparinized fresh chicken blood, washed and suspended in PBS at 1% (V/V). Firstly, safety doses of Sur and Pig were determined by incubating it with erythrocyte suspension for 2 h, centrifugating and measuring the supernatant, compared to the untreated erythrocyte wells, at 490 nm. Then, serial dilutions of safe dose (3 mg/ml) of Sur and Pig were incubated with an equal volume of H1N1 (8 hemagglutination units). After 1 h incubation, These mixtures of Sur-H1N1 or Pig-H1N1 were incubated with an equal volume of 1% chicken erythrocyte suspension in PBS in 96-well plate for 1 h before observing the aggregation of erythrocytes.

#### Inhibitory potential on neuraminidase activity

The activity of neuraminidase was determined according to instructions of fluorometric-blue neuraminidase assay kit (Abcam, USA). Briefly, serially-diluted concentrations of Sur or Pig were incubated with NeuroBlue indicator and neuraminidase for 100 min at 37 °C and fluorescence intensity (extension/emission) was measured at 320/450 nm using spectrofluorometer (BMG LabTech, Germany).

### Investigation of anti-coronavirus activity against HCoV-229E and SARS-CoV-2

#### Evaluating anti-HCoV-229E potential

##### Determination of inhibiting potency on cytopathic activity of HCoV-229E

Vero E6 cells were seeded, into a 96-well culture plate, at a density of 2 × 10^4^cells/well. After cell attachment (24 h), then the culture medium in 96 well plate was replaced with 100 µl of diluted HCoV-229E suspension containing CCID50 (10^–3^). Then serially-diluted concentrations of Sur or Pig were added to wells, excluding positive infected (untreated) control wells. After 72 h incubation of culture plates at 37 °C in 5% CO_2_, 0.03% crystal violet solution was added as showed above. The IC_50_ was calculated by the Graphpad Prism software.

### Investigation of anti-HCoV-229E action modes

The modes of anti-HCoV-229E action were conducted as previously described [16]. To evaluate the virucidal effect, serially-diluted concentrations of Sur were incubated with HCoV-229E for 1 h before being added to monolayer of Vero E6 cells. The anti-adsorption effect was assessed by pre incubating cells for 1 h with serially-diluted concentrations of Sur, then discarding this medium and incubating with HCoV-229E inoculum for 1 h. In the anti-replicative effect, serially-diluted concentrations of Sur were incubated with HCoV-229E-infected Vero E6 cells for 1 h. In these three conditions, the infected medium was replaced by new culture medium on cells. Following 72 h, all cells were stained with crystal violet, as demonstrated above, for estimation of IC_50_ by the Graphpad Prism software.

### Evaluating anti-SARS-CoV-2 potential by determinating SARS-CoV-2 main protease activity

3-Chymotrypsin protease assay was performed using 3CL Protease, Untagged (SARS-CoV-2) assay kit (BPS Bioscience, USA). Briefly, serially-diluted concentrations of Sur or Pig were pre-incubated with 3CL protease (0.5 µg/ml) for 30 min, then the reaction was started by the addition of 3CL protease substrate (40 µM). After 2 h of incubation, fluorescence intensity was measured at excitation 360 nm and emission 460 nm.

### Determinaton of scavening activity against radical species-mediated lung damage

The anti-radical potentials of Sur and Pig were detected against superoxide anion, hydroxyl radical and nitric oxide using colorimetric assays. Superoxide anion radical (O_2_^·¯^) scavenging activity was evaluated, according to method described by Ravishankara et al. [20] by incubating serially-diluted concentrations of Sur or Pig with a reaction mixture of 67 mM phosphate buffer (pH 7.8), EDTA, 1.5 mM nitroblue tetrazolium, 1.5 mg% NaCN, and 0.12 mM riboflavin for 15 min. Then the absorbance was measured at 530 nm. Hydroxyl radical scavenging (^•^OH) activity of Sur or Pig was detected by incubation their serially-diluted concentrations with 9 mmol/L salicylic acid, 9 mM FeSO_4_ and 9 mM H_2_O_2_ for 1 h at 37 °C. The absorbance was then measured at 510 nm [21]. Nitric oxide radical (NO) scavenging activity was quantified using Griess reagent [22]. The Graphpad Prism software estimated the concentrations (IC_50_) of Sur and Pig at which free radicals were scavenged by 50%.

### Molecular docking studies

#### Structures acquisition and preparation

The available three-dimensional crystal structures of H1N1 Influenza virus neuraminidase and SARS-CoV-2 3CL protease were obtained from the Protein Data Bank (PDB, www.rcsb.org) PDB IDs: (PDB ID: 6HP0 and PDB ID: 7VTH [23]**,** respectively) handled with the Molecular Operating Environment (MOE) software package version MOE 2019.102, Chemical Computing Group, Montreal, Canada Unwanted ligands and residues were removed*.* The structures of surfactin and C50 carotenoid bacterioruberin were prepared and refined employing the default “Structure preparation” MOE setting, then energy minimized employing Amber10: EHT force field with reaction-field electrostatics (an interior dielectric constant of 1 and an exterior dielectric of 80) using an 8–10 Å cutoff distance.

#### Docking simulations

The prepared surfactin and C50 carotenoid bacterioruberin were docked into the ligand binding sites by the Molecular Operating Environment (MOE) software package version MOE 2019.102, Chemical Computing Group, Montreal, Canada [14]. using Alpha HB and the Triangular matcher algorithm as scoring and placement functions creating the top 100 non-redundant poses of the lowermost binding energy conformers. Docking was showed with induced fitting protocol. Results were estimated as S-scores with RMSD value < 2.5 Å. The molecular interactions graphical representations were inspected and generated.

### Statistical analysis

The collected data was statistically analyzed using Tukey-Post Hoc multiple comparison one-way ANOVA and unpaired *t-tests* (SPSS 16). All data were demonstrated as mean ± standard error of the mean (SEM). A *P* value of ≤ 0.05 was considered significant.

## Results

### The biosurfactant and pigment recovery

The archaeon *Natrialba* sp. M6 was isolated, cultivated and identified as mentioned previously in the method section by [8, 9]. The Sur and the Pig were extracted according to the abovementioned methods and the dried produced Sur and Pig were obtained to be ready for the subsequent experiments. It is worth noting that, the chemical structure of the partially purified extracted Pig of *Natrialba* sp. M6 was previously accredited to C50 carotenoid bacterioruberin according to obtained spectroscopy (Raman, FT-IR, & Nuclear magnetic resonance) and spectrometry (GC–mass, LC–mass) results (Hegazy et al. [8]). Additionally, the recovered *Natrialba* sp. M6 biosurfactant extract was deduced to be a cationic lipopeptide according to GC–MS results, compositional analysis content relationship and Zetasizer as described by Hegazy et al. [9]. Further analysis for the Sur extract was carried out through HPLC against a cyclic lipopeptide (Surfactin) as standard.

### HPLC of the extracted biosurfactants of *Natrialba* sp. M6

**Fig. 1:**
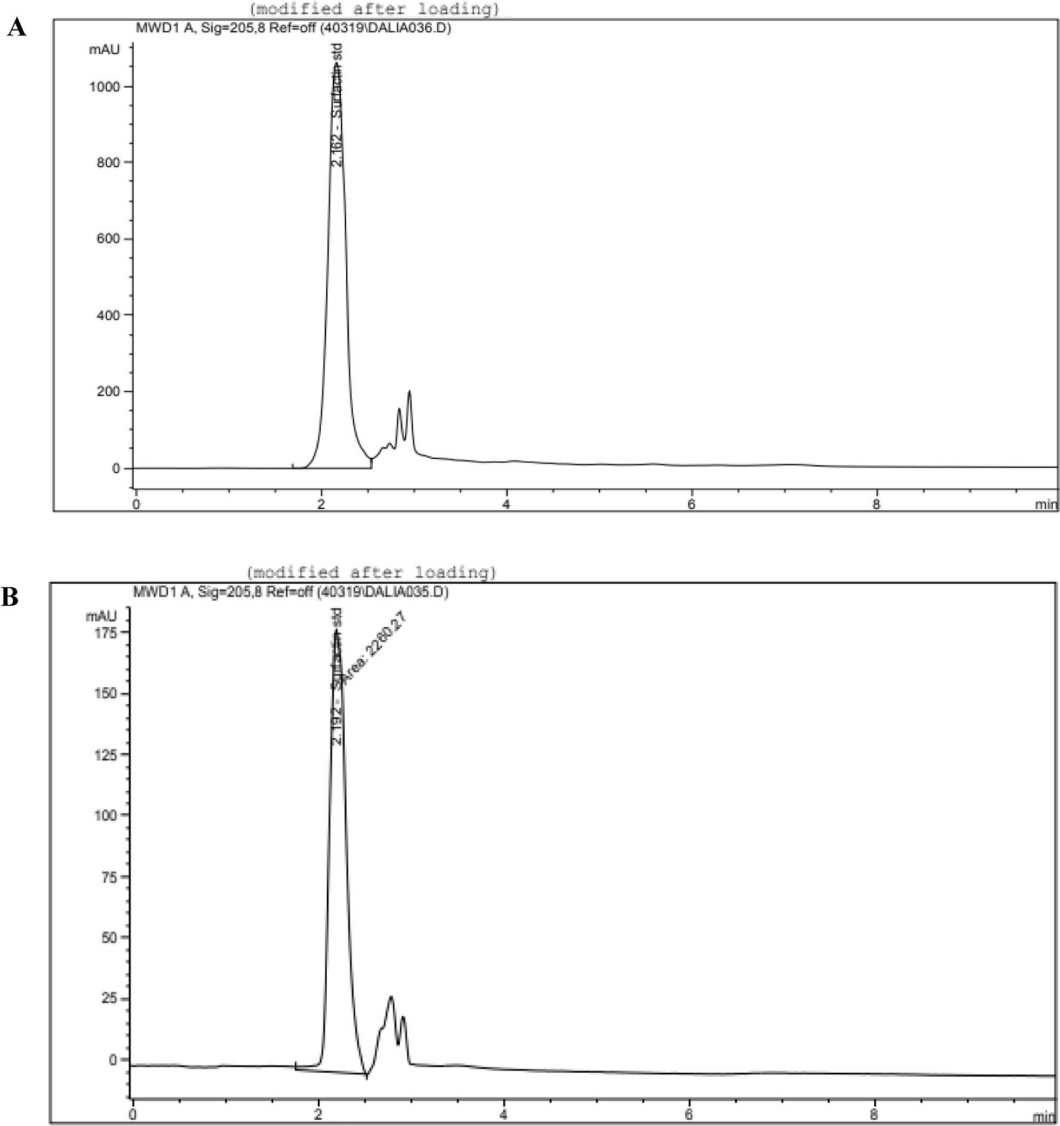
HPLC analysis of surfactin standard and extracted Sur molecules. The separation patterns of **A** surfactin standard and **B** sample of the extracted biosurfactant at 2.162 min and 2.192 min, respectively

The Surfactin retention time (RT) of the standard and the sample (Sur) during HPLC were 2.162 min and 2.192 min, respectively (Fig. 1A, B), the similarity between the recorded RT of the extracted biosurfactant produced by *Natrialba* sp. M6 and Surfactin standard RT confirmed the presence of surfactin molecule(s) in the extracted crude biosurfactant.

### Anti-pulmonary viral activity with unveiling the mode of action(s)

**Fig. 2:**
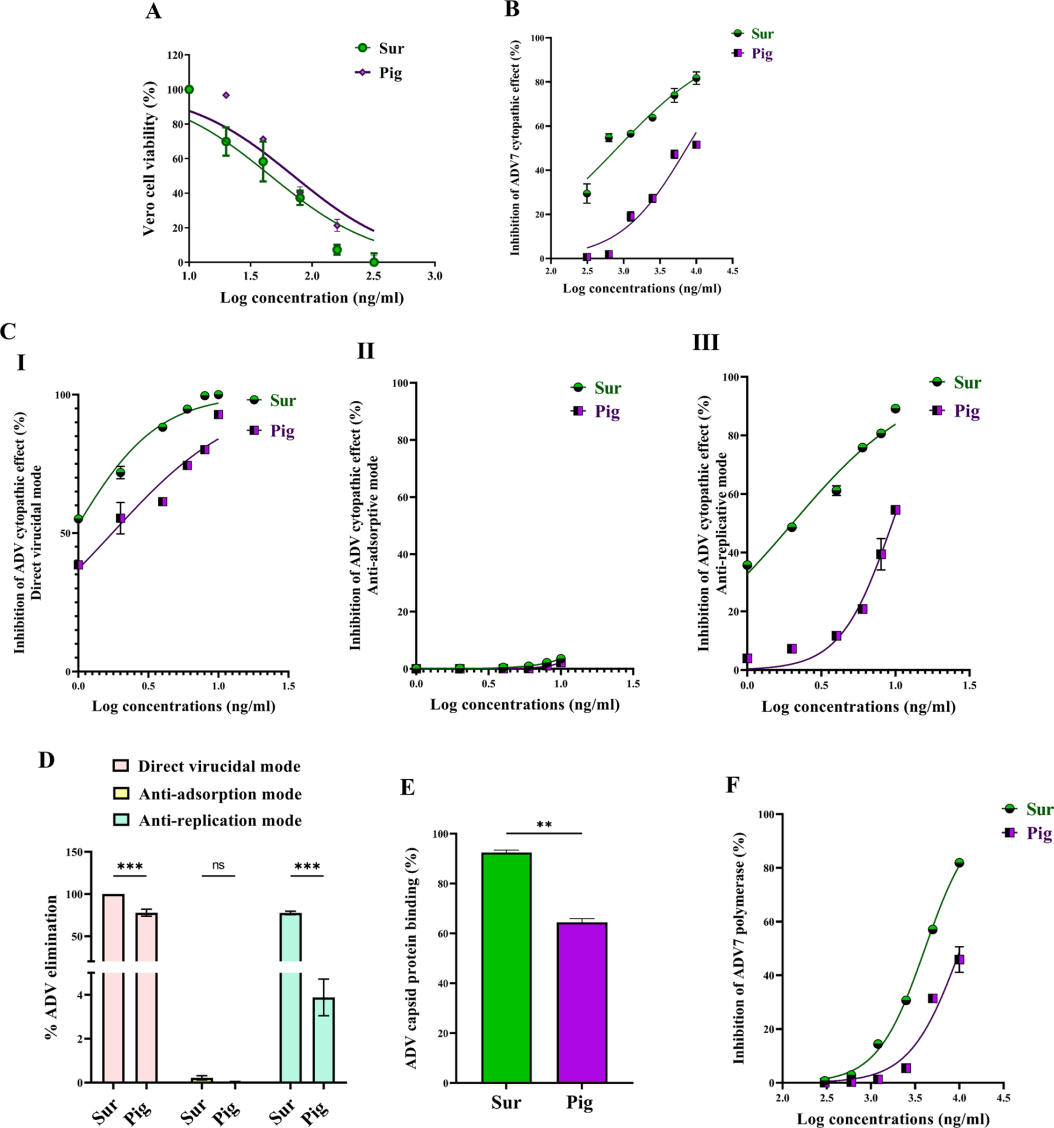
The cytotoxicity and anti-adenoviral effects of biosurfactant (Sur) and carotenoid pigment (Pig). **A** Viability of adenovirus host cell (Vero) after 72 h incubation with serially-diluted concentrations of Sur or Pig. **B** The cell lysis inhibition % after treatment ADV-infected Vero cells with serially-diluted concentrations of Sur or Pig. **C** The inhibitory potency (%) of Sur and Pig on cytopathic effect of ADV in three studied modes, including (I) direct virucidal, (II) anti-adsorption, and (III) anti-replication activities. **D** QPCR assessment of viral elimination in three studied action modes. The unveiling direct virucidal activity by illustrating **E** their ADV capsid protein binding percentage and anti-replicative activity by demonstrating **F** their inhibition potency on ADV polymerase

**Fig. 3:**
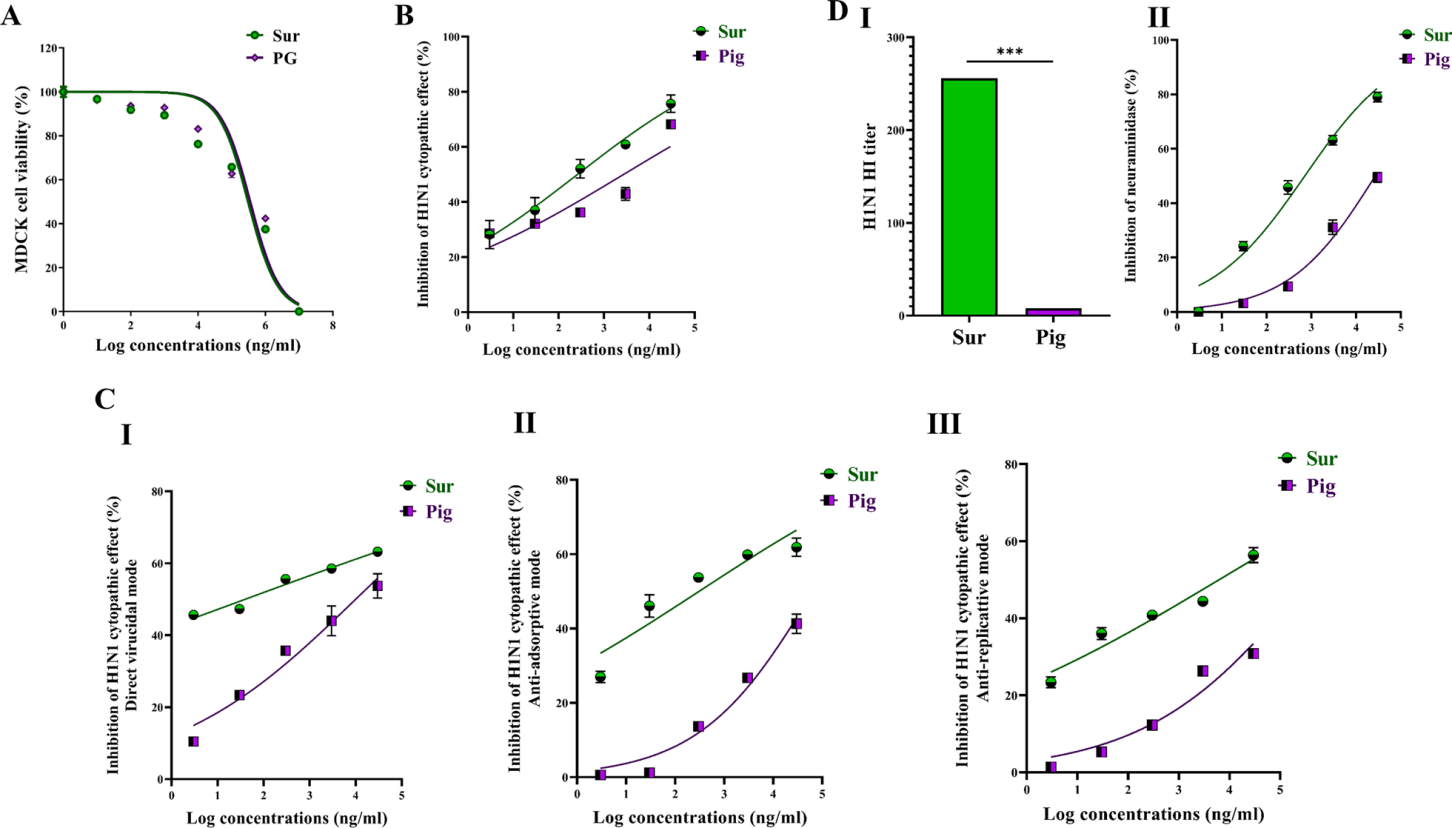
The cytotoxicity and anti-H1N1 effects of biosurfactant (Sur) and carotenoid pigment (Pig). **A** Viability of host cell (MDCK) of H1N1 after 72 h incubation with serially-diluted concentrations of Sur or Pig. **B** The cell lysis inhibition % after treatment H1N1-infected MDCK cells with serially-diluted concentrations of Sur or Pig. **C** The inhibitory potency (%) of Sur and Pig on cytopathic effect of H1N1 in three studied modes, including (I) direct virucidal, (II) anti-adsorption, and (III) anti-replication activities. **D** The unveiling their direct virucidal activity by illustrating (I) hemagglutination titer and (II) the inhibition percentage of neuraminidase

Prior investigating the antiviral activity of the isolated Sur and Pig, their cytotoxicity against viral host cells (Vero, MDCK, and Vero E6) was important to assess. Dose response curves (Figs. 2A, 3A, 5A) were used to estimate EC_50_ values of Sur that were 206.11, 304.4, and 412.5 μg/ml, respectively, and Pig were 251.9, 354.5, and 153.7 μg/ml, respectively. Also, it was found that the estimated EC_100_ values of Sur were 9.847, 30.82, and 32.20 μg/ml, respectively and Pig were 17.41, 39.98, and 44.49 μg/ml, respectively. At their corresponding safe doses, the following anti-pulmonary viral activities were investigated.

### Anti-ADV7 activity

Figure 2B illustrates that both Sur and Pig can suppress cell lysis effect of ADV7 on Vero cells, in a dose-dependent manner. Sur exhibited higher anti-ADV7 activity than Pig, as illustrated by a lower IC_50_ value of Sur (0.814 ± 0.08 μg/ml) than Pig (7.388 ± 0.39 μg/ml) for 50% inhibiting ADV7 cytopathic activity. Additionally, IC_50_ values were calculated from dose response curves (Fig. 2C I–III) of three different antiviral protocols by directly incubating different concentrations of Sur or Pig with ADV7 (direct virucidal), incubating the same dilutions of Sur or Pig with Vero cells before ADV7 infection (anti-adsorption), and addition of these doses of Sur or Pig to the infected cells (anti-replication). The IC_50_ values of their virucidal and anti-replicative modes (≤ 10 μg/ml) were the lowest when compared to anti-adsorption. Both Sur and Pig showed very weak anti-adsorption activity (< 4%) for ADV7 (Fig. 2C II). In terms of IC_50_ values, Sur exhibited stronger virucidal and anti-replicative activities (2.969 ± 0.516 and 3.571 ± 0.078 μg/ml) than that of Pig (4.476 ± 0.376 and 10.06 ± 0.118 μg/ml).

Importantly, their anti-ADV7 mode was investigated at 3 μg/ml, by qPCR quantifying viral load, in the three following cases: direct incubating with ADV7, adding to Vero cells prior to viral infection, and placing on ADV7-infected Vero cells (Fig. 2D). The highest viral inactivation (99.98 ± 0.03% and 77.82 ± 2.39%) was recorded in the case of direct virucidal effect of Sur and Pig, respectively. Furthermore, Sur exhibited higher anti-replicative potential by eliminating 77.65 ± 1.20% of ADV7 than Pig (3.88 ± 0.48%). Sur and Pig did not reveal significant anti-adsorptive activity (≤ 0.2%) as shown in Fig. 2D. The main anti-ADV7 action mode (direct virucidal) of Sur and Pig was more thoroughly investigated by assessing their binding percentages to ADV7 capsid protein that were 92.45 ± 0.66% and 64.50 ± 1.01%, respectively (Fig. 2E). The second most anti-ADV7 activity (anti-replicative) of Sur was also thoroughly quantified as IC_50_ for ADV polymerase inhibition (4.091 ± 0.05 μg/ml) which was significantly lower than Pig (10.48 ± 0.95 μg/ml) as illustrated in Fig. 2F.

### Anti-H1N1 activity

Both Sur and Pig can inhibit H1N1 cytopathic activity at IC_50_ of 0.267 ± 0.06 μg/ml and 2.73 ± 0.23 μg/ml, respectively, in dose-dependent manner (Fig. 3B). Furthermore, IC_50_ values were estimated from dose response curves of three antiviral modes by direct incubation of serially-diluted concentrations of Sur or Pig with H1N1 (direct virucidal), incubating the same dilutions with MDCK cells prior to H1N1 infection (anti-adsorption), and adding these serial doses to the infected cells (anti-replication) as shown in Fig. 3C I–III. The IC_50_ values of their virucidal mode (≤ 10 μg/ml) were found to be the lowest when compared to the other two modes (anti-adsorption and anti-replication, respectively). In terms of IC_50_ values, Sur had higher virucidal, anti-adsorptive, and anti-replicative activities (0.043 ± 0.01, 0.317 ± 0.05, and 8.093 ± 0.30 μg/ml) than that of Pig (10.23 ± 1.22, 0.317 ± 0.05, and 380.7 ± 34.8 μg/ml). For more declaration of the two most anti-H1N1 mechanisms, the inhibition potency for H1N1 hemagglutinin binding and neuraminidase activity were detected. Firstly, the safety of Sur and Pig was assessed on chicken RBCs which were used as host cells in the hemagglutination (HA) assay. It was found that up to 3 mg/ml of each did not show any toxicity on RBCs. Figure 3D I declares that Sur had a 32-fold higher HA titer than Pig for inhibition of H1N1 hemagglutinin binding to RBCs. Sur also showed a lower IC_50_ (0.716 ± 0.04 μg/ml) than Pig (27.05 ± 2.58 μg/ml) for inhibiting neuraminidase activity (Fig. 3D II).

### Docking into H1N1 Influenza virus neuraminidase active site

**Fig. 4:**
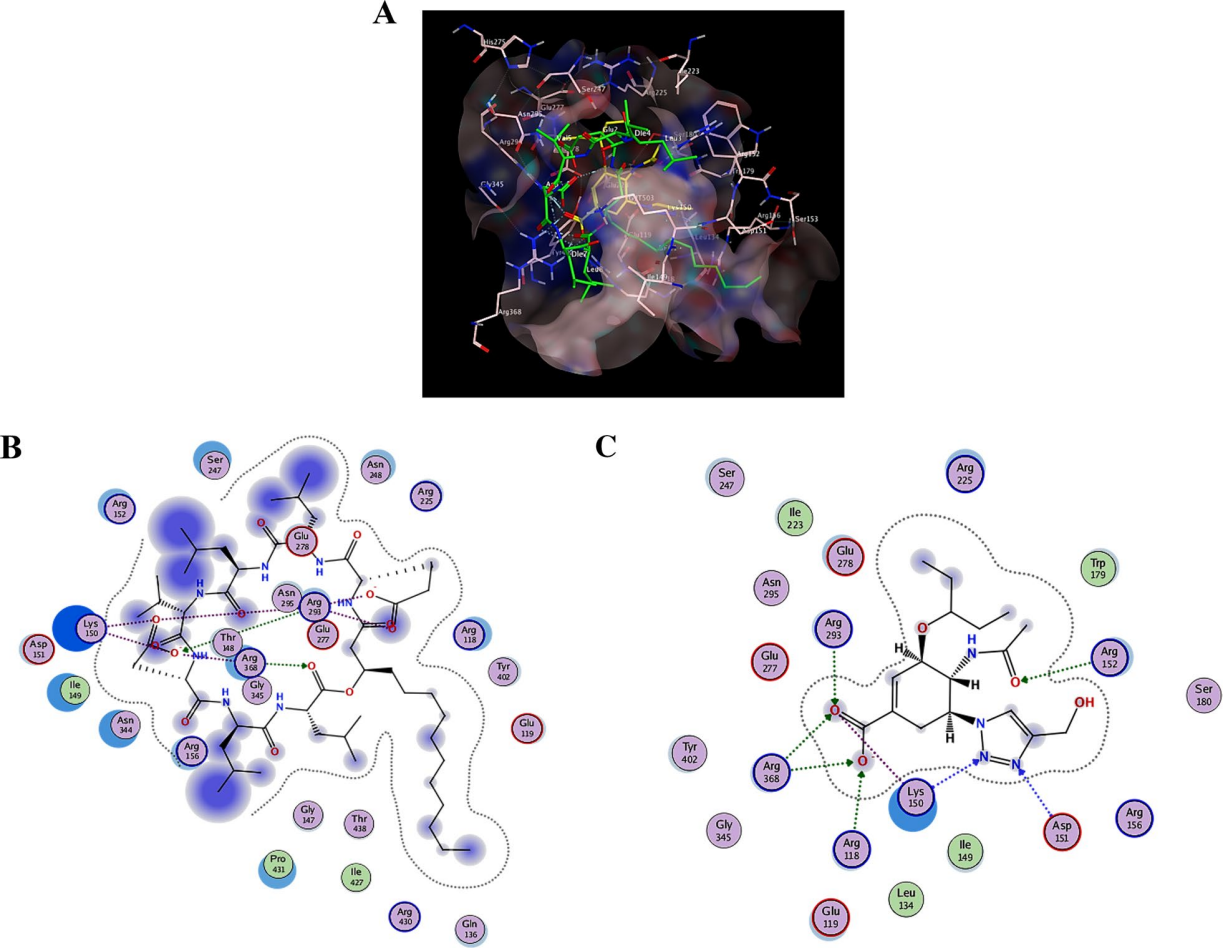
Molecular docking analysis for interaction with H1N1 Influenza virus neuraminidase. **A** 3D binding mode of surfactin (green sticks) into the co-crystallized ligand (yellow sticks) binding site (molecular surface), **B** 2D interactions of surfactin, and **C** 2D interactions of the co-crystallized ligand within the prepared crystal structure of H1N1 Influenza virus neuraminidase (PDB ID: 6HP0)

Herein, the complex of H1N1 Influenza virus neuraminidase in complex with oseltamivir triazole derivative was obtained from the Protein Data Bank (PDB ID: 6HP0 [15]**.** After the unwanted residues were eliminated, the structural coordinates were prepared employing the Molecular Operating Environment (MOE) software package version MOE 2019.102 [14]**,** utilizing its default protocol. Docking of surfactin into the co-crystallized ligand binding site (Fig. 4A, B) demonstrated good fitting with binding affinity (ΔG = − 9.48 kcal/mol). Hydrogen bonding interactions were detected between the viral Arg293 and Arg368 with Asp6 and Leu8 of surfactin, respectively. Molecular docking of C50 carotenoid bacterioruberin failed to produce acceptable inferred binding free energy with the active site of neuraminidase.

### Anti-coronaviral activity against HCoV-229E and SARS-CoV-2

#### Anti-HCoV-229E potential

**Fig. 5:**
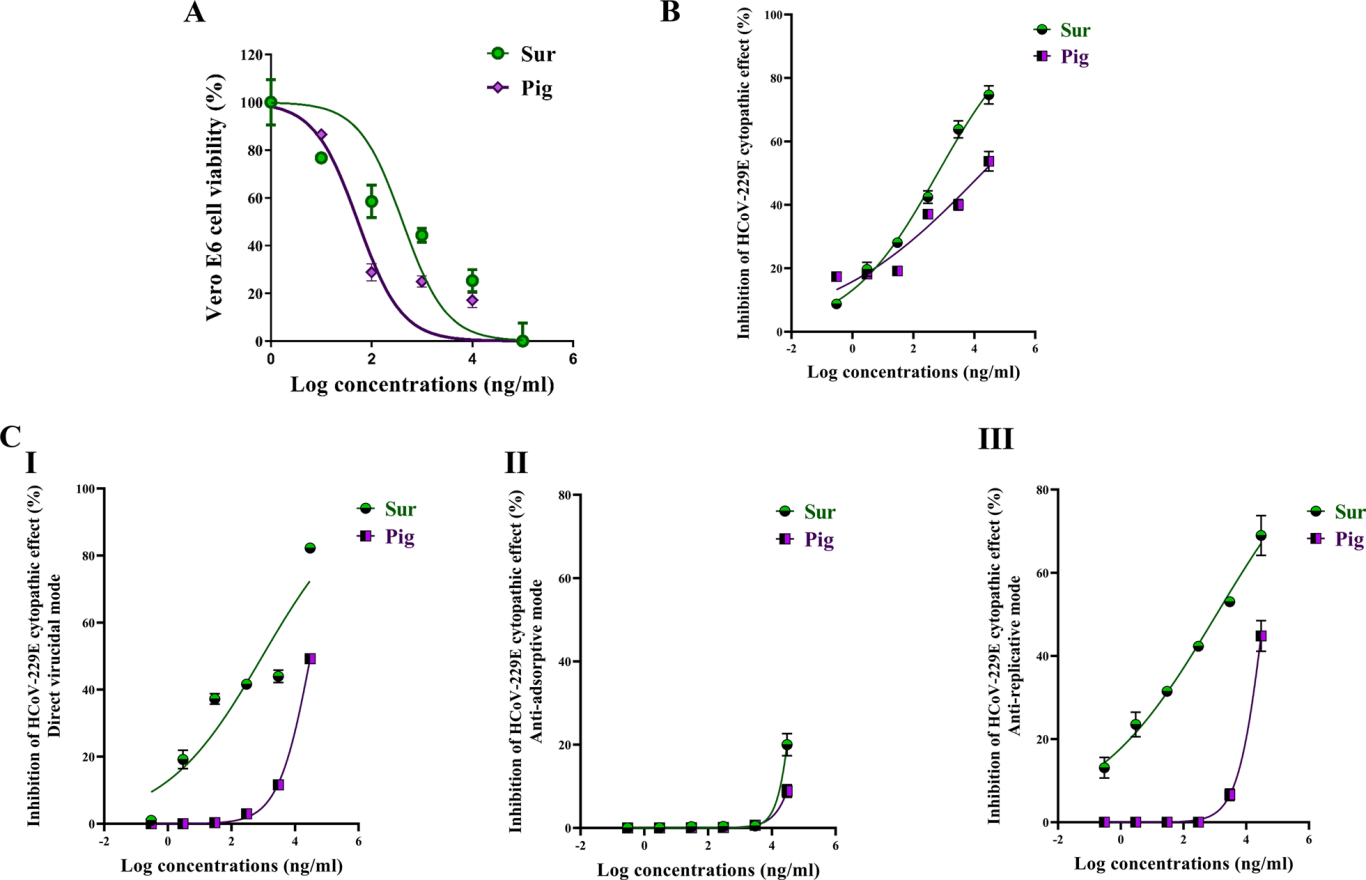
The cytotoxicity and anti-HCoV-229E effects of biosurfactant (Sur) and carotenoid pigment (Pig). **A** Viability of host cell (Vero E6) of HCoV-229E after 72 h incubation with serially-diluted concentrations of Sur or Pig. **B** The cell lysis inhibition % after treatment HCoV-229E-infected Vero E6 cells with serially-diluted concentrations of Sur or Pig. **C** The inhibitory potency (%) of Sur and Pig on cytopathic effect of HCoV-229E in three studied modes, including (I) direct virucidal, (II) anti-adsorption, and (III) anti-replication

As demonstrated in Fig. 5B, Sur had a stronger dose-dependent inhibition of HCoV-229E cytopathic potential than Pig, as evidenced by 240-fold lower IC_50_ of Sur (0.636 ± 0.03 μg/ml) than Pig (144.4 ± 11.2 μg/ml). By investigating three potential modes of actions, it was found that both samples exhibited direct virucidal properties on extracellular infectious particles and anti-replication activities without detectable anti-adsorption potential against HCoV-229E (Fig. 5C I–III). More importantly, Sur exhibited significantly higher virucidal and anti-replicative activities at lower IC_50_ (0.952 ± 0.027 and 1.194 ± 0.227 μg/ml, respectively) than Pig (31.22 ± 0.80 and 37.18 ± 3.63 μg/ml, respectively).

#### Anti-SARS-CoV-2 potential

**Fig. 6:**
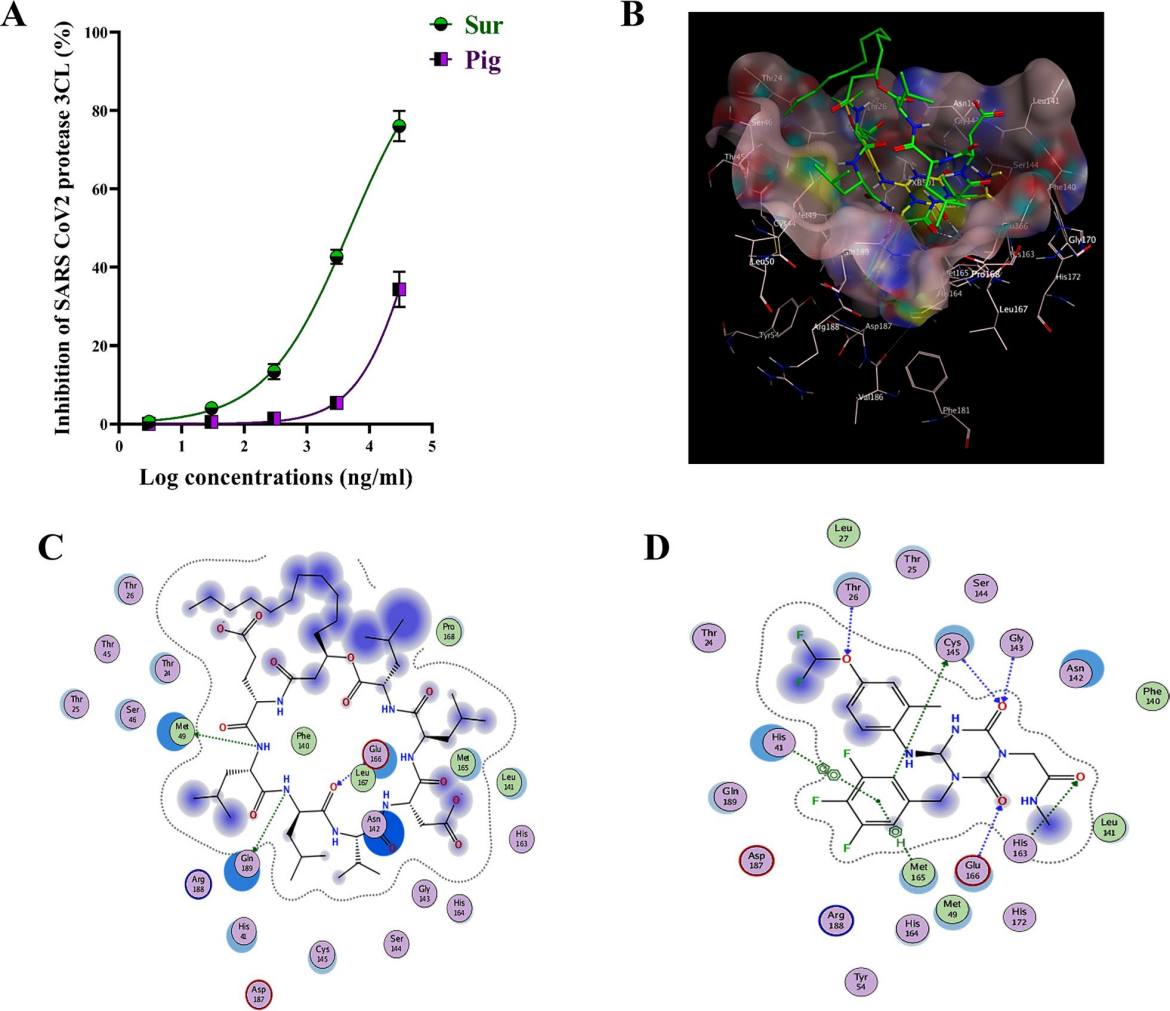
The inhibition potential on SARS-CoV-2 in the term of suppressing 3-chymotrypsin like (3CL) protease activity with illustrating molecular docking analysis. **A** Inhibition percentages of biosurfactant (Sur) and carotenoid pigment (Pig) for 3CL protease activity. **B** 3D binding mode of surfactin (green sticks) into the co-crystallized ligand (yellow sticks) binding site (molecular surface), **C** 2D interactions of surfactin, and **D** 2D interactions of the co-crystallized ligand within the prepared crystal structure of SARS-CoV-2 3CL protease (PDB ID: 7VTH)

The potent virucidal and anti-replicative modes of Sur against coronaviruses were deeply investigated by assessing its repressing potency for 3CL protease of SARS-CoV-2, compared to Pig (Fig. 6A). Sur inhibited 3CL protease more effectively in a dose-dependent manner, with a lower IC_50_ (5.319 ± 0.305 μg/ml) than Pig (69.30 ± 7.98 μg/ml).

### Docking into SARS-CoV-2 3CL protease active site

**Fig. 7:**
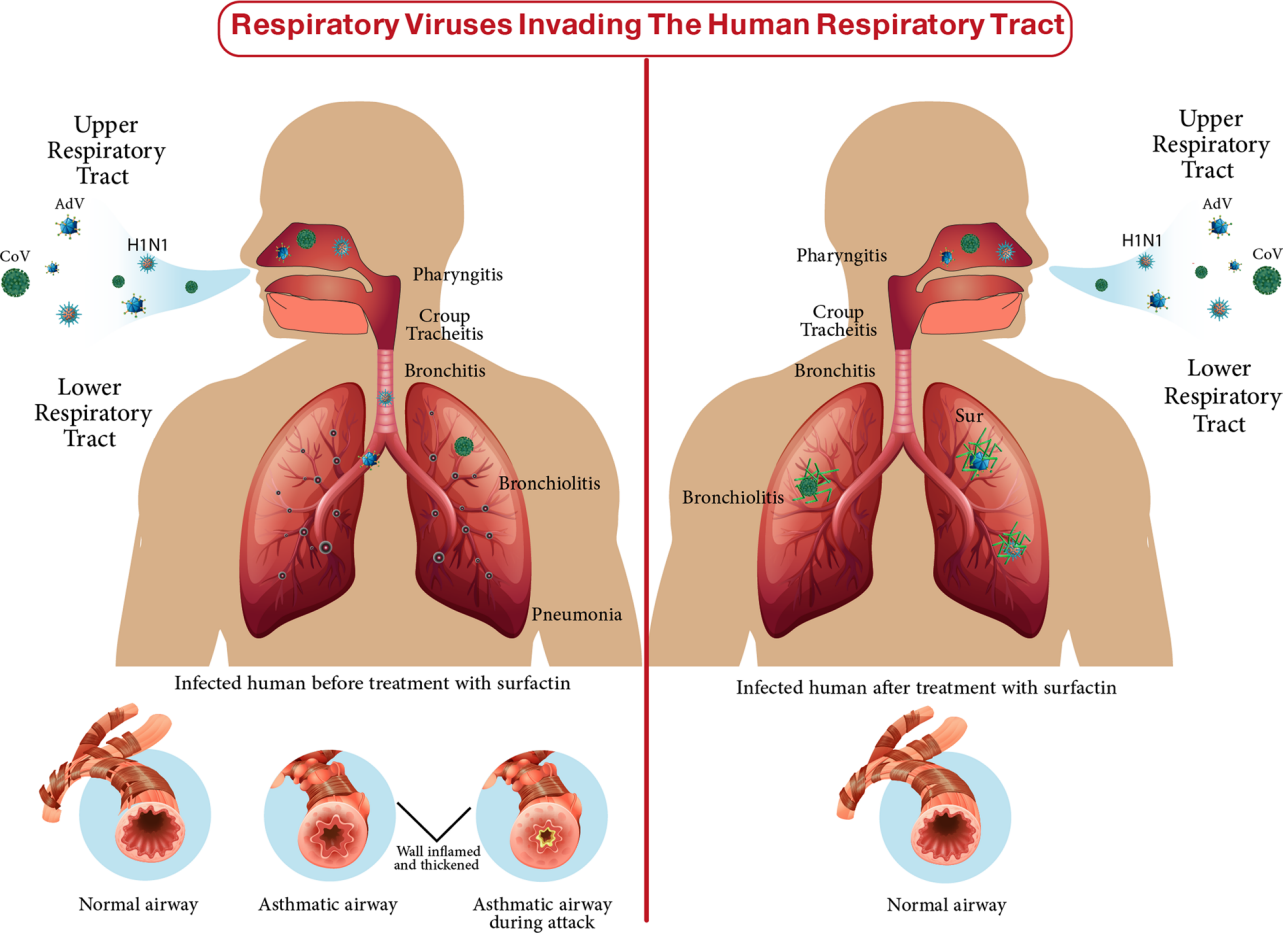
The respiratory viruses (CoV, AdV and H1N1) invading the human respiratory system before and after the treatment with the produced surfactin

In this inferred docking model, the SARS-CoV-2 3CL protease complexed structural coordinates with a non-covalent inhibitor were retrieved from the Protein Data Bank (PDB ID: 7VTH ^11^. The unwanted residues were removed and eliminated, then the preparation of the structure was prepared using the Molecular Operating Environment (MOE) software package version MOE 2019.102 [14] default protocol. The docking results in Fig. 6B, C and D showed that surfactin has the ability to bind with the active site of the virus with promising binding affinity (ΔG = − 9.37 kcal/mol) displacing the bonding interactions of hydrogen with the key residue Met49, Gln189 and Glu166 through both L- and D-Leucine residues of surfactin. On the other hand, C50 carotenoid bacterioruberin failed to fit into the active site at acceptable binding free energy. Figure 7 shows the respiratory viruses (CoV, AdV and H1N1) invading the human respiratory tract. These respiratory viruses have invaded the human respiratory system. The invasion refers to the viruses entering and replicating within the respiratory cells, leading to the onset of infection and associated symptoms such as pneumonia disease, However, after the application of surfactin as a treatment, the effects on these respiratory viruses are observed.

### Radical scavenging activity

**Fig. 8:**
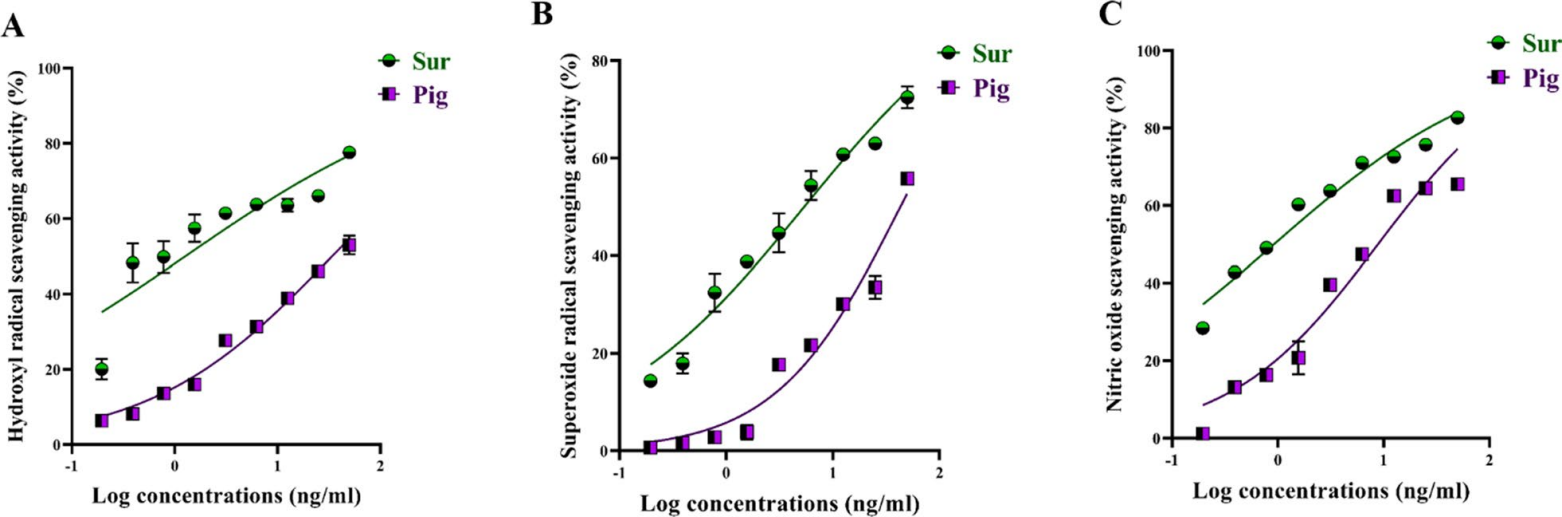
Antioxidant activity of biosurfactant (Sur) and carotenoid pigment (Pig) via scavenging potential of **A** hydroxyl, **B** superoxide, and **C** nitric oxide radicals

Due to crucial role of virus-induced free radical storm in damaging the infected host cell, the radical scavenging is an essential therapeutic antiviral strategy. As shown in Fig. 8A–C, Sur efficiently scavenged ONRS in dose-dependent manner when compared to Pig. The concentrations of Sur required to scavenge 50% of superoxide anion, hydroxyl radical, and nitrogen oxide were 1.283, 5.401, and 0.920 μg/ml, respectively, significantly lower than the corresponding IC_50_ doses of Pig (34.02, 43.03, and 8.846 μg/ml).

## Discussion

Finding a potent alternative supportive broad treatment for pulmonary viral infections is critical to prevent ARDS, severe oxidative damage, and pulmonary failure, which are the main causes of death. According to our recent studies, Sur and Pig extracts of haloalkaliphilic archaeon *Natrialba* sp. M6 are promising antiviral treatments [8, 9]. Thus, it is important to figure out the antiviral activity of Sur extract (mainly surfactin, as evidenced by HPLC result “Fig. 1”) as well as Pig extract against the studied pulmonary viruses by providing insight into their mechanisms of action.

The current study provides clear evidences that the anti-pulmonary viral potential of archaeal compounds, at their safe doses, is primarily through direct virucidal and/or anti-replicative modes. Also, it showed superior antiviral efficacy of the extracted Sur, compared to Pig, in the term of direct virucidal and anti-ADV replication via binding to viral capsid protein and inhibiting ADV’s DNA polymerase activity, respectively (Fig. 2). Recent finding, via competitive saturation experiment and transmission electron microscopy imaging, illustrated that novel zwitterionic surfactant micelles interacts with ADV5 by binding with its hexon capsid protein during film matrix-forming process [24]. Previous study revealed Sur’s potential to bind to capsid protein of Norovirus [25]. Regarding another anti-ADV mechanism involving DNA polymerase inhibition, our recent studies demonstrated that Sur suppressed DNA polymerase of herpes simplex at 4.39 µg/mL and Pig inhibited DNA polymerase of HBV at 4.99 µg/mL [8, 9]. Based on literature, this study is the first to investigate the anti-ADV potency of Sur and Pig and revealing their possible mode of action(s). Regarding influenza viruses (e.g., H1N1), this infection causes pulmonary surfactant dysfunction, leading to ARDS [12]. Lung surfactant proteins inactivate influenza viruses through mediating the innate immune response, binding to hemagglutinin oligosaccharides, and inhibiting neuraminidase [26]. Although neuraminidase inhibition is the current treatment of choice for influenza viruses, there are issues that drug (neuraminidase inhibitors)-resistant influenza viruses can emerge in the future and spread to high-risk populations [27, 28]. Therefore, additional anti-H1N1 activity via hemagglutinin binding is critical for efficient virucidal potential. As shown in the current findings, Sur exhibited higher anti-H1N1 potential, not only for neuraminidase inhibition but also for hemagglutinin binding, than Pig (Fig. 3). Pulmonary surfactant competitively binds to HIN1-hemagglutinin, promoting hemagglutinin aggregation with disrupting viral functions, and subsequently halting its entry and replication [29–31]. Previous studies found that surfactant lipid components (palmitoyl-oleoyl-phosphatidylglycerol and phosphatidylinositol) suppressed H1N1 and H3N2 infection by inhibiting their binding to the host plasma membrane with attenuating viral gene expression and thereby their replications [27, 32]. A unique previous investigation demonstrated potent anti-hemagglutination potential of surfactant protein D against 31 various influenza virus strains, including H1N1 [33]. Another study reported that surfactant protein D inhibited hemagglutination activity via binding to globular domain of hemagglutinin [34]. Docking of surfactin and C50 carotenoid into the co-crystallized ligand binding site of H1N1 viral glycoprotein resulted in an inferred model with notable binding affinity (ΔG = − 9.48 kcal/mol). Interactions by hydrogen bonding were noticed between the viral amino acids Arg293 and Arg368 with Asp6 and Leu8 of surfactin, respectively. C50 carotenoid failed to detect acceptable binding free energy with the active site. Coronaviruses (particularly, SARS-CoV-2) remain a serious risk for humans around the world. Thus, anti-viral drugs are both safe and effective are highly envisaged. The current results revealed that Sur and Pig had potent direct virucidal and anti-replicative effects on HCoV-229E as well as high inhibition potential on 3CL protease of SARS-CoV-2. In the terms of these mentioned parameters, Sur exhibited stronger anti-coronaviruses potential than Pig (Figs. 5, 6). A recent study reported that SARS-CoV-2 suppressed the production of pulmonary Sur [35], implying that supplementation with external Sur could be a promising treatment for this extremely risky virus. Another recent study found that Sur, from *Bacillus subtilis,* reduced the infectivity of different coronaviruses (HCoV-229E, MERS-CoV, SARS-CoV, and SARS-CoV-2) by disrupting the integrity of virion envelope (direct virucidal activity) leads to inhibiting the binding of viral spike protein to host cell membrane receptor [36]. Additionally, Sen et al. (2022) performed molecular docking analysis that revealed multiple hydrophobic and hydrogen bond interactions between Bacillus surfactins and 3CL protease [37]. Finding a safe and broad-spectrum therapeutic compound capable of inhibiting both coronaviruses’ entry and replication is extremely needed. According to our knowledge, this is a pioneer study in investigating the inhibitory impact of *Natrialba* sp. M6 extracts on SARS-CoV-2 main protease, which is attractive therapeutic strategy for halting coronavirus replication. Docking results indicated that the extracted surfactin was able to bind the active site of SARS-CoV with promising binding affinity (ΔG = − 9.37 kcal/mol) showing interactions of hydrogen bonding with the key residue Met49, Gln189 and Glu166 through both L- and D-leucine residues of surfactin. On the other hand, C50 carotenoid bacterioruberin failed to fit into the active site of the virus at acceptable binding free energy. Importantly, the scavenging potency of Sur and Pig against free radical species-mediated viral complications was illustrated. In the term of IC_50_, Sur exhibited a stronger scavenging potential for ONRS than Pig (Fig. 8). Radical species are closely responsible for the uncontrolled inflammation-mediated lung damage in pulmonary viral infection, including SARS CoVs [38, 39]. Coronaviruses inhibit ACE2 activity leads to accumulating angiotensin II which stimulates the generation of ONRS via activating NADPH oxidase. The generated ONRS oxidize cysteine residues of ACE2 and RBD, resulting in increased affinity of SARS- CoVs to this host receptor as well as activating proinflammatory cytokines, subsequently increase severity of COVID [38, 40]. Accordingly, antioxidant supplementation provides adjuvant tool to fight viral infection and pathological complication [38, 39]. It is known that carotenoids attenuate oxidative stress and regulate the overactivation of inflammatory cytokines and proinflammatory enzymes, which are caused by viral infection [11, 12]. Supplementation of natural Sur exhibited potent antioxidant and anti-inflammatory activities and showed improvement in lung function and in numerous lung diseases (pneumonia and asthma) [12, 13, 41].

Taking all shown evidences together, this current study states the promising efficacy of Sur of archaeon *Natrialba* sp. M6 against pulmonary viruses, including ADV, H1N1, HCoV-229E, and the highest risk coronavirus (SARS-CoV-2), via direct virucidal and/or anti-replicative modes, compared to Pig. Additionally, this study demonstrated Sur’s antioxidant potency, which is essential for halting viral infection complications. These findings are considered preliminary experiments for the next preclinical study, which will include a pharmacokinetic analysis of Sur and a more in-depth investigation for its antiviral mechanisms.

## Data Availability

All data produced during this study are included in this published article and the datasets used and/or analyzed during the current study are available from the corresponding author on reasonable request.

## Abbreviations

ADV: Adenovirus
ELISA: Enzyme-linked immunosorbent assay
FTIR: Fourier
GC–Mass: Gas chromatography–mass spectrometry
H1N1: Haemagglutinin type 1 and Neuraminidase type 1
HPLC: High-performance liquid chromatography
Kcal: Kilocalorie
NMR: Nuclear magnetic resonance
ONRS: Oxygen and nitrogen radical species
Pig: C50 carotenoid pigment
SARS-CoV-2: Severe Acute Respiratory Syndrome Coronavirus 2
Sur: Surfactin
